# Superstructures of chiral nematic microspheres as all-optical switchable distributors of light

**DOI:** 10.1038/srep14183

**Published:** 2015-09-24

**Authors:** Sarah J. Aβhoff, Sertan Sukas, Tadatsugu Yamaguchi, Catharina A. Hommersom, Séverine Le Gac, Nathalie Katsonis

**Affiliations:** 1 Laboratory for Biomolecular Nanotechnology (BNT), MESA+Institute for Nanotechnology, University of Twente, PO Box 217, 7500 AE Enschede, The Netherlands; 2 BIOS, Lab on a Chip Group, MESA+Institute for Nanotechnology and MIRA Institute for Biomedical Engineering and Technical Medicine, University of Twente, PO Box 217, 7500 AE Enschede, The Netherlands.

## Abstract

Light technology is based on generating, detecting and controlling the wavelength, polarization and direction of light. Emerging applications range from electronics and telecommunication to health, defence and security. In particular, data transmission and communication technologies are currently asking for increasingly complex and fast devices, and therefore there is a growing interest in materials that can be used to transmit light and also to control the distribution of light in space and time. Here, we design chiral nematic microspheres whose shape enables them to reflect light of different wavelengths and handedness in all directions. Assembled in organized hexagonal superstructures, these microspheres of well-defined sizes communicate optically with high selectivity for the colour and chirality of light. Importantly, when the microspheres are doped with photo-responsive molecular switches, their chiroptical communication can be tuned, both gradually in wavelength and reversibly in polarization. Since the kinetics of the “on” and “off” switching can be adjusted by molecular engineering of the dopants and because the photonic cross-communication is selective with respect to the chirality of the incoming light, these photo-responsive microspheres show potential for chiroptical all-optical distributors and switches, in which wavelength, chirality and direction of the reflected light can be controlled independently and reversibly.

Since the discovery of liquid crystals in the late 19^th^ century, spherical microspheres of liquid crystals have been investigated experimentally. Otto Lehmann reported as early as 1904 on the study of para–azoxyanisole microspheres, with the aim to decipher the nature of the liquid crystalline order^1^. More recently, the emergence of soft photonics has triggered a renewed interest into microspheres of liquid crystals, as combining the birefringence of the liquid crystal with a spherical topology gives rise to unprecedented optical properties^2^,^3^,^4^. These special optical properties provide new opportunities in terms of optical vortex generation^5^, omnidirectional lasing^6^, chiral sorting^7^, and photonic cross-communication^8^,^9^.

A major obstacle towards the development of new technologies based on liquid crystal microspheres lies in controlling their size and size distribution, which is also essential to control and harness their internal structure, as size and structure are intimately correlated for confined liquid crystals^10^. Recent progresses in microfluidics, specifically, the emergence of droplet microfluidics have addressed this challenge by providing platforms enabling the large-scale production of micrometer-size objects with well-defined sizes, shapes, and narrow size distributions^4^,^11^,^12^. In this context, producing microspheres of chiral nematic (cholesteric) liquid crystals shows particular potential as cholesteric liquid crystals reflect light selectively: only a narrow range of wavelengths is reflected, with a specific polarisation. Under a polarized light microscope, an isolated chiral nematic microsphere with parallel surface anchoring shows a single reflection spot located at the core of the sphere and centred around a wavelength λ(0) = n.p where n is the mean refractive index and p the pitch of the cholesteric helix. The optical properties of isolated chiral nematic microspheres with planar surface anchoring have been described before^6^,^7^,^26^. These selective reflection properties confer cholesteric microspheres with potential applicability as optical couplers for microfluidic systems, autonomous sensors, and building blocks for high-security identification tags because highly complex optical patterns can emerge when the microspheres are assembled as monolayers^8^ or bilayers^13^.

Close-packed monolayers of chiral nematic microspheres can be formed by simple self-assembly of microspheres, provided these microspheres display a sufficiently narrow size distribution. In chiral nematic microspheres with planar surface anchoring, the helix propagates radially in all directions^13^,^14^,^15^,^16^. As a consequence, a microsphere illuminated from the top can reflect light of different colours, however each colour is only reflected in a specific direction. The colour of the central reflection spot is centred around λ(0) = n∙p whilst the cones of light reflected in the periphery are characterised by shorter wavelengths. For example, for the part of the droplet where the helical axis is tilted at an angle of θ = 45° with respect to the incoming light beam, the reflected light will be directed towards the plane of the self-assembled microspheres, with equal intensity along all directions. This property provides superstructures of chiral nematic microspheres with potential applicability as distributors of light, routing specific wavelengths uniformly towards a certain direction of the sample. Conventional hybrid distributors and switches are slow and require more energy because of the conversion from light to electricity (and conversely). This is why all-optical distributors are becoming very appealing systems to develop data transmission further. However, their potential applicability remains limited by their lack of versatility: most all-optical distributors remain beam splitters, with restricted capabilities since they typically split the light into two sub-beams only, or guide the light in a single pre-defined direction^17^.

Here, we design a superstructure of self-assembled chiral nematic microspheres that can distribute incoming light in any direction, while displaying selectivity with respect to polarity of the incoming light. In this structure, we also include light-responsive microspheres that operate as switches within the superstructures. These switching units allow controlling the colour and polarisation of light, which is reflected selectively for a desired direction of reflection. In so doing, we demonstrate that the wavelength and polarization of light, on one hand, and the angle of reflection, on the other hand, can be decoupled within these photonic superstructures. Using light as an external trigger to switch the distribution of light beams offers remote, temporal and local controllability and consequently constitutes an attractive alternative to conventional hybrid distributors of light.

## Results and Discussion

### Preparation of chiral nematic microspheres in a microfluidic setup

**Figure 1:**
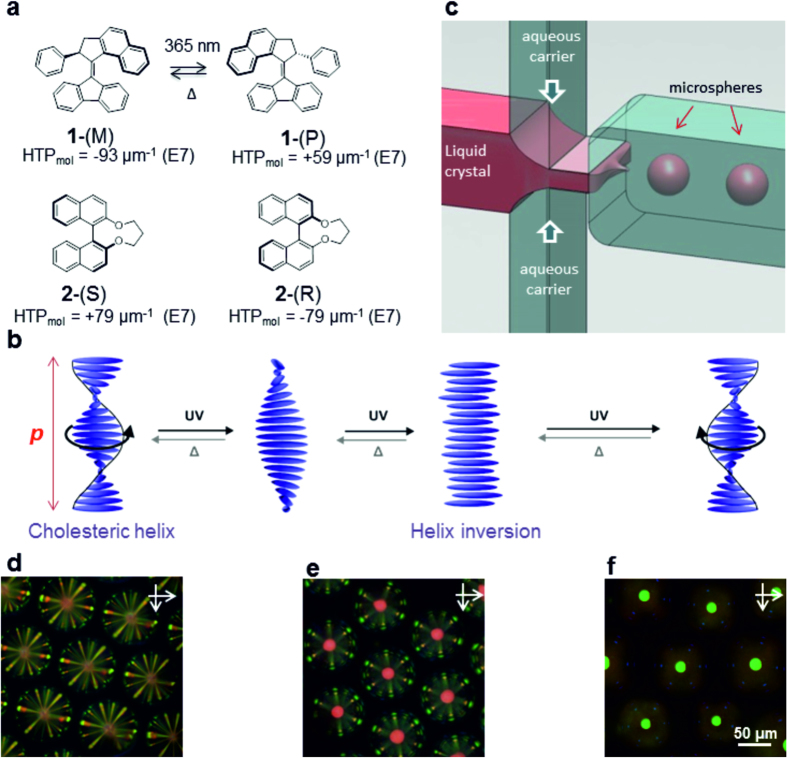
Generation of chiral nematic microspheres and their assembly into superstructures. (**a**) Light-responsive molecular dopant **1**-(M), its photo-isomer **1**-(P) and shape-persistent chiral dopants **2**-(S) and **2**-(R). The values of helical twisting powers (HTP) are given for the nematic host E7. (**b**) Schematic representation of a photo-responsive chiral nematic liquid crystal. The pitch p corresponds to a full helix turn. (**c**) The microfluidic set-up. The microspheres are dispersed in an aqueous carrier solution containing a surface agent that promotes planar surface anchoring of the liquid crystal molecules. (d-f) Microscopy images of microsphere superstructures under crossed polarizers (**d**) Close-packed superstructure of microspheres doped with **2**-(S). (**e**) Close-packed superstructure of microspheres doped with **2**-(R). (**f**) Close-packed superstructure of microspheres doped with **1**-(M).

Photo-controllable cholesteric liquid crystals are typically designed by doping a nematic liquid crystal with a few percent light-responsive molecular switches^18^, for example the light-responsive molecules shown Fig. 1a. Upon irradiation with light, photo-isomerisation of the molecular switches induces an increase of the cholesteric pitch^18^,^19^ (Fig. 1b). Here, microspheres of light-responsive cholesteric liquid crystals were prepared by doping a conventional nematic liquid crystal (E7) with a light-responsive molecular motor **1**-(M), at a concentration of 4.9 wt%. In the dark, the resulting cholesteric liquid crystal is characterised by a pitch p = 350 nm and a reflection band centered around λ(0)  = 530 nm at normal incidence.

We prepared passive chiral nematic microspheres by doping E7 with 3.8 wt% of shape persistent chiral dopants **2**-(R) and its enantiomer **2**-(S), which are both well-known as efficient dopants for a range of nematic liquid crystals^20^,^21^,^22^. The resulting chiral nematics are respectively left-handed (LH) and right-handed (RH), and are both reflecting in the red at normal incidence, with reflection bands centred around λ_LH_ (0)  = 715 nm and λ_RH_ (0)  = 700 nm.

The microspheres were produced at room temperature using a dedicated microfluidic set-up consisting of a glass/silicon microchip and a MFCS Fluigent system for fine control of the flow rates in the device (Fig. 1c). Specifically, the liquid crystal was introduced in a flow-focussing configuration with a (1:1) glycerol/water carrier solution supplemented with 3 wt% polyvinylalcohol (PVA) (Fig. 1c). PVA ensures a planar orientation of the liquid crystal molecules at the interface with the carrier. Upon deposition from the carrier solution onto glass slides, and following partial evaporation of the carrier, self-assembly of the microspheres into hexagonal, two-dimensional, closely packed superstructures was observed. Optical microscopy confirmed that the microspheres are monodisperse (Fig. 1d–f). Their diameters were fine-tuned by adjusting the two flow rates, and they typically ranged from 150 μm to 180 μm (Table S1).

### Chiroptical communication in a hexagonal array of microspheres

**Figure 2:**
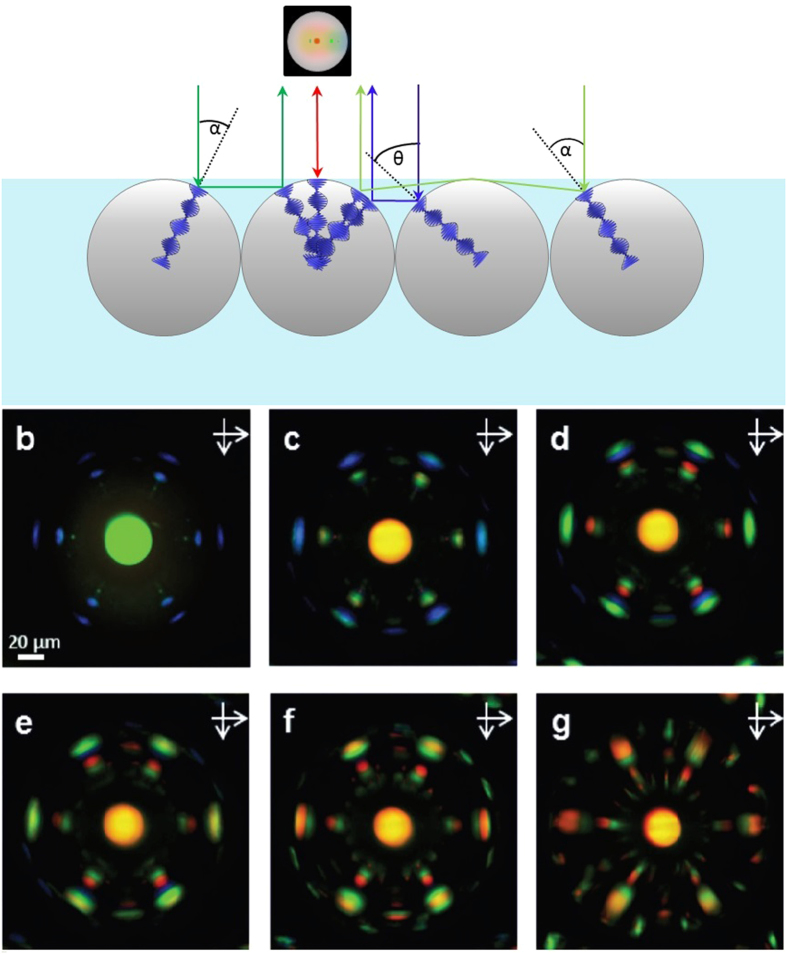
All-optical tuning of the cross-communication pattern in a hexagonal superstructure of microspheres. (**a**) The mechanism of cross-communication. The pathways leading to the central reflection is represented with red arrows, while the blue and green arrows show the reflections due to direct and indirect communication, respectively. The scheme above the central microsphere shows its appearance when observed under the microscope with crossed polarizers. (**b**–**g**) The snapshots taken with crossed polarizers represent one photo-responsive microsphere doped with **1**-(M), embedded in a close-packed hexagonal array of similar microspheres. Upon activation with UV light for 2 min the dopant is converted into **1**-(P), which induces the tuning of the central reflection colour and the pattern of photonic interaction. Images were taken at 3s (**b**), 23s (**c**), 43s (**d**) 63s d), 83s (**e**), 103s (**f**), 123s (**g**) of UV irradiation.

A homogeneous superstructure of photo-responsive microspheres doped with **1**-(M) was prepared, and its collective optical response to illumination with white light was investigated. Figure 2a represents a side view of these microspheres, once assembled at the air/carrier interface. The microspheres present a green central reflection accompanied by blue reflection spots pointing towards their six neighbours (Fig. 2b). These peripheral reflections arise from chiroptical communication between the microspheres^8^. At normal incidence, a certain wavelength λ(0) = n∙p is reflected upwards and appears as a central reflection spot (Fig. 2a, red arrow). Depending on the geometry of the microsphere at the incidence point of light, the angle θ between the incoming light beam and the axis of the cholesteric helix varies between 0° and 90°. For θ = 45° the incoming light is reflected towards the nearest neighbour of the microsphere, giving rise to a cross-communication signal (blue arrow). For θ < 45° the incoming light is reflected towards the air/carrier interface, where it is totally internally reflected in an angle α = −θ and hits the nearest neighbour or one of the next nearest neighbours which will then reflect the light back up (green arrows). For θ > 45° the light is reflected towards the nearest neighbour but from there it cannot be reflected back up. These different pathways for light give rise to the observed complex cross-communication pattern.

The microspheres doped with **1**-(M) respond to the ultraviolet component of the incoming light. Upon isomerisation of **1**-(M), the cholesteric helix unwinds (Fig. 1b), which induces a red shift of the central reflection spot. Moreover, this colour shift is accompanied by a complete rearrangement of the cross-communication pattern (Fig. 2b–g). Interestingly, this intricate photo-induced pattern shows a higher level of complexity than in other previously reported arrays of liquid crystal microspheres. This difference is likely due to the fact that previous investigations involved the self-assembly of microspheres trapped in between two glass slides, a design that is restricting the cross-talk to direct communication^9^. In contrast, in the superstructure presented here, the microspheres self-assemble at the air/carrier interface, thereby enabling indirect communication mediated by total internal reflection also. Increased complexity in the photo-induced optical pattern demonstrates clear potential in terms of applications to high-security identification tags. Using these light-responsive superstructures, dynamic patterns could be created with a fine control over the number and location of cross-communication spots simply by extending the illuminated area. In addition, the wavelength could be controlled dynamically and independently within the complete spectrum of light (including the invisible range of UV- and infrared light) and the chirality of the reflected light could be controlled as well. Mixed superstructures composed by photo-active, photo-passive or even non-cholesteric microspheres, would lead to patterns being as unique as easy to generate.

### Chiral nematic microspheres as chiroptical distributors and switches

**Figure 3:**
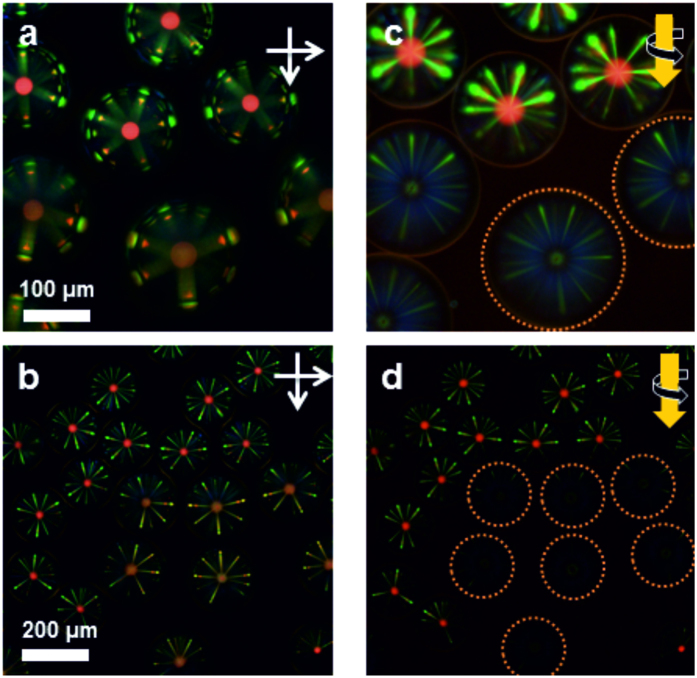
Mixed superstructure of chiral nematic microspheres doped with 1-(S) and 1-(R), as a chiroptical distributor of light. (**a**,**b**) Under crossed polarizers, both types of microspheres are visible clearly. (**c**,**d**) Selective visualization of RH microspheres by illumination with RH circular polarized light. The LH microspheres become almost invisible (they are circled in orange as a guide to the eyes).

Combining right-handed (RH) and left-handed (LH) microspheres allows the preparation of a heterogeneous superstructure where chiroptical cross-communication between microspheres of opposite chirality can be investigated, provided they have comparable sizes. Such a mixed superstructure is shown Fig. 3. Under crossed linear polarizers, both series of microspheres display a full cross-communication pattern, comparable to the one observed for homogeneous arrays (Fig. 3a,b). In contrast, under irradiation with right handed circularly polarized white light, both the central reflection and the peripheral colours of the LH microspheres embedded in a network of RH microspheres switch off (Fig. 3c,d). The disappearance of the central reflection spot (θ = 0°) is attributed to the selective reflection of chiral nematic liquid crystals, and is thus in agreement with the fact that cross-communication is based on selective reflection. More surprisingly, we observe that the reflection is only highly selective in polarization for θ = 0°. When θ 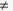
0°, the reflection involves more complex polarization modes^23^,^24^, that subsist even under irradiation with right handed light (Fig. 3c), although they are much less intense. Nevertheless these results indicate that the cross-communication between microspheres of opposite chirality is switched off under illumination with circularly polarised light and prove that the photonic cross-communication is selective with respect to the chirality of the incoming light—a result that opens up exciting perspectives in terms of chiroptical switching in all-optical distributors.

**Figure 4:**
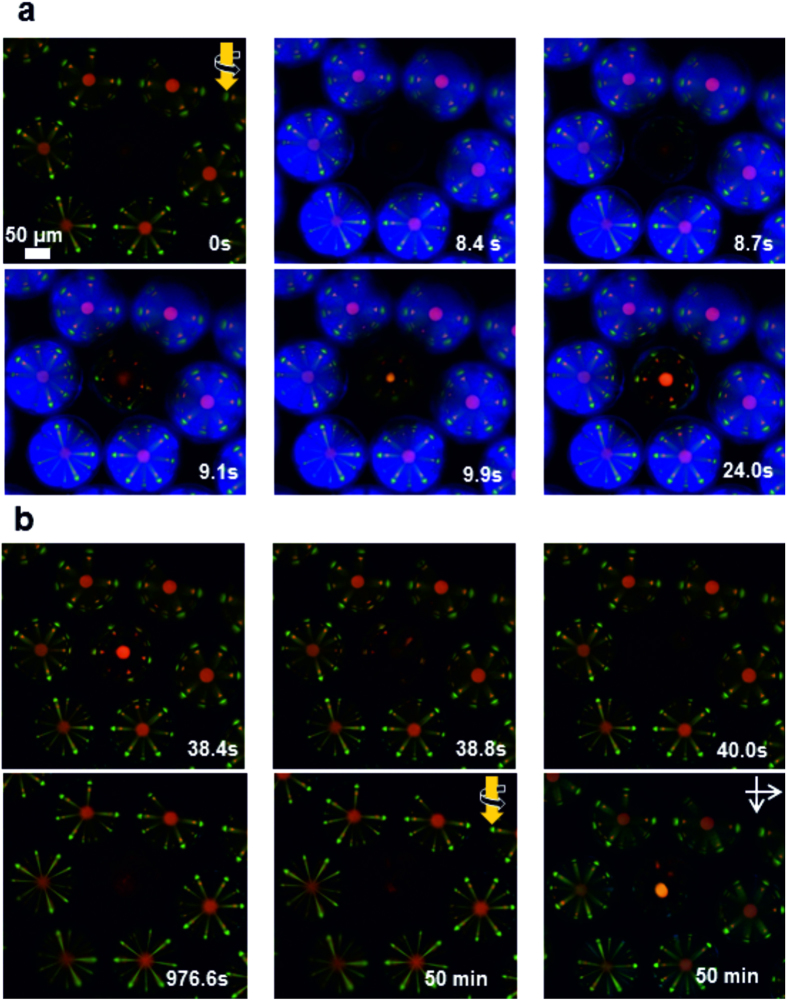
Photo-responsive chiral nematic microspheres as chiroptical switches for distributors of light. (**a**) A photo-responsive microsphere doped with **1**-(M) is inserted in a superstructure of LH microspheres and the resulting array is observed under crossed polarizers. The central photoactive microsphere is not visible initially. After 30 s of irradiation with UV light, the central reflection appears, accompanied with photonic cross-communication. (**b**) After irradiation with UV light is stopped, the photo-responsive microsphere relaxes and the reflection signals disappear. The last two images are taken after about 50 min of relaxation using circular polarized light and linear polarized light respectively. For linear polarized light the reflection signals of all microspheres are visible.

Based on the chiroptical selectivity of the cross-communication pattern, we designed a switching unit for the cross-communication between microspheres. To demonstrate the switchable functionality, a mixed network was designed out of RH microspheres doped with **2**-(S) and photo-responsive microspheres doped with **1**-(M). Under right-handed circularly polarised light, no communication is established between the two series of microspheres because the photo-responsive microspheres are left-handed in their stable state (Fig. 4). Alternatively, under irradiation with UV light, the handedness of the photo-responsive microspheres is inverted, and both series of microspheres start to communicate, as evidenced by their reflection pattern (Fig. 4a). After irradiation with UV light is stopped, the cholesteric helix relaxes back to equilibrium, which translates into a continuous change in the intensity of the reflection pattern, and eventually its disappearance under illumination with right-handed light (Fig. 4b). In comparison to the experiment displayed in Fig. 2, the reflection pattern in Fig. 4 does only change in intensity, because only the central microsphere undergoes structural changes, whereas the neighbouring spheres remain unaffected. The neighbours reflect light of a certain wavelength and handedness in a certain angle towards the central microsphere. This central microsphere changes the pitch and handedness of its cholesteric helix under UV light. Only when the pitch and handedness enable the central microsphere to reflect light that matches the light coming from the neighbours, there is cross-communication between those microspheres. The colour of the reflection spots can therefore not change because it solely depends on the pitch of the neighbouring spheres that do not respond to light. Also the number and position of the spots are not affected because the geometry of the array as well as the angle of reflection of the neighbouring spheres do not change either. The handedness of the cholesteric helix instead inverts, which we use in this experiment to switch on and off the photonic cross-communication. Noticeably, the photo-step is completed after about 30 s, *i.e.*, it is much faster than their relaxation in the dark, that takes over 50 min to complete. Interestingly, the relaxation kinetics can be adjusted easily, either by modifying the intensity of irradiation or by molecular engineering of the photo-responsive dopant^25^. Overall, these results demonstrate that photo-responsive chiral nematic microspheres are controllable and reversible switching units that are selective for both the wavelength and polarisation of light.

### *In-situ* formation of chiral nematic microbeads

The chiral nematic microspheres exhibit excellent stability over several months as long as they are kept in the carrier solution. The assemblies of microspheres, however, show limited stability of up to several hours due to evaporation of the carrier solution, that increases the surface tension and leads to their deformation over time. One option to alleviate evaporation consists in parking the microspheres between two glass slides^9^, although that alternative restricts the cross-talk to direct communication only, and consequently reduced the complexity of the pattern drastically. Another alternative consists in stabilising the microspheres by using *in-situ* polymerization. Earlier investigations have reported on stabilizing liquid-crystalline droplets permanently by using *in-situ* polymerization^26^. However, the cholesteric order was not preserved well. Here, we stabilize the cholesteric microspheres by using only partial *in-situ* polymerization in order to minimize the disruption of the liquid crystalline order, and with the potential advantage to preserve the dynamic properties of the droplets also.

**Figure 5:**
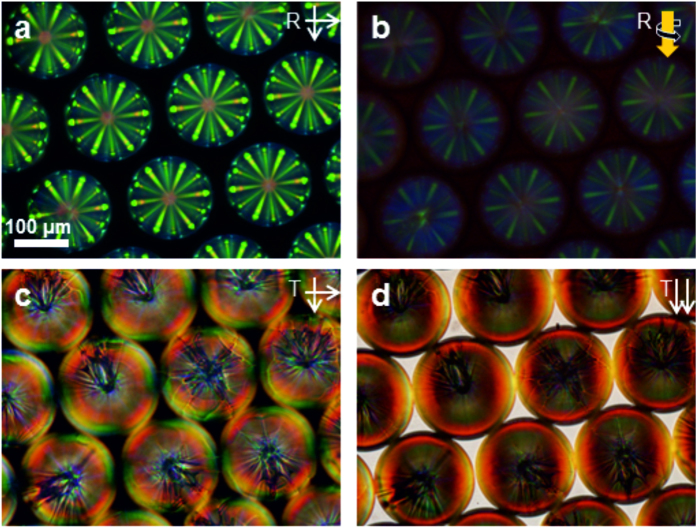
Polymer-stabilized chiral nematic microbeads. The chiral nematic order, provided by doping with **2**-(S), is mostly preserved after polymerization and the microspheres do not deform after the carrier solution has evaporated. (**a**) Reflected light under crossed polarizers. (**b**) Reflected right-handed circular polarized light. (**c**) Transmitted light under crossed polarizers. (**d**) Transmitted light under parallel polarizers.

We generated microspheres of a chiral nematic doped with **2**-(R) supplemented with 5 wt% of a cross-linking monomer and traces of photoinitiator that absorbs in the visible range (Fig. S1). The microspheres were produced in the same microfluidic approach under exclusion of light and collected together with the carrier solution in a small vial. A fraction of the emulsion was subsequently deposited on a glass slide, and after their self-assembly into a hexagonal superstructure, the microspheres were polymerized under UV light. One day after polymerisation, optical microscopy revealed that their spherical shape was preserved and, more importantly, that the cross-communication pattern between the microbeads exhibited only minor changes (Fig. 5a,b). Specifically, the red central reflection is less intense, which could be an indication of a red-shifting of the cholesteric pitch following polymerization – a phenomenon that has been observed upon polymerisation of thin films, and attributed to either in-plane switching or stretching of the cholesteric helix due to tilting of reactive mesogenes away from the director during cross-linking^27^.

Next to the increased stability of these chiral objects, we observe that the liquid crystalline order is partially disrupted upon *in-situ* polymerization, and consequently the special optical properties of the microbeads is not preserved perfectly (Fig. 5c,d). We attribute this introduction of disorder to the fact that polymerization is initiated in topological defect areas preferentially. In a well-organized cholesteric droplet with parallel surface anchoring and sufficiently high radius-to-pitch ratio, the expected texture is the so-called “onion texture”. This texture is characterized by one radial defect^3^. We observed that the organization in close proximity of the defect is disrupted due to internal stresses imposed by the polymer chains. We therefore conclude that polymerization preferentially occurs in the vicinity of this radial defect. Because the overall organization of the droplet is preserved by polymerization, the typical optical signature of the droplets is preserved also. However, the presence of topological defects in liquid crystal microspheres being unavoidable, we conclude that *in-situ* polymerization is not likely to provide chiral nematic beads that are the perfect counterpart of chiral nematic droplets in terms of organisation. Other strategies to freeze the chiral nematic order have been implemented earlier in thin films^28^ and could be considered as alternatives.

## Conclusion

We have generated monodisperse chiral nematic microspheres using a continuous flow approach, with fine control on the size distribution, using a dedicated silicon/glass microfluidic system. We could polymerise these chiral objects while preserving their shape, in order to form polymer-stabilised microspheres of cholesteric liquid crystals. Beyond all-optical distributors and switches, these stabilised microbeads will reveal instrumental for applications ranging from chiral sorting^7^ to biomimetic materials^29^.

We find that two-dimensional, closely packed, hexagonal superstructures that are formed by self-assembly of chiral nematic microspheres exhibit complex cross-communication that is revealed simply by observation under crossed polarisers. The geometry of the cross-communication pattern originates from the geometry of the superstructure; specifically, hexagonal superstructures of microspheres give rise to hexagonal patterns of reflection. Using different sizes of monodisperse microspheres in one layer could yield binary superstructures with unprecedented reflection patterns. Materials based on binary lattices of protein-coated polymer microspheres have shown high promises for sensor applications^30^ and their development could benefit from the dynamic photonic properties that we report on. Moreover, we show that photonic cross-communication between objects of opposite chirality can be switched on and off, by irradiation with circularly polarised white light, which highlights that cross-communication is selective with respect to the chirality of the incoming light. Next we demonstrate that cross-communication between the microspheres can be switched on and off reversibly, by using photo-responsive liquid crystals. These results confirm that chiral nematic microspheres are promising building blocks for the design of all-optical switchable distributors of light. Importantly for their applicability to data communication, the selective reflection of these building blocks can be adjusted to virtually cover the whole spectrum of light—including infrared. Our results consequently demonstrate the potential of photo-responsive microspheres of liquid crystals as chiroptical switches and distributors in future all-optical data transmission technologies.

## Methods

### Synthesis of the dopants

Light-responsive molecular dopant **1** was synthesized following a procedure adapted from the literature^31^,^32^. Subsequently, the two enantiomers were separated by chiral HPLC on a CHIRALPAK AD-H column using a methanol/ethanol (1:1) mobile phase, affording >99% ee of **1**-(M). The synthesis of the shape-persistent chiral dopants **2**-(S) and **2**-(R) was carried out as described earlier^22^.

### Preparation of the cholesteric mixtures and carrier solution

E7 is a commercially available nematic liquid crystal (Merck) (Fig. S1), that is liquid crystalline at room temperature. It was dissolved in dichloromethane and mixed with 3.8 wt% **2**-(S), 3.8 wt% **2**-(R) or 4.3 wt% **1** to provide right-handed (RH), left-handed (LH) and a photo-responsive, initially left-handed chiral nematic liquid crystal, respectively. Dichloromethane was evaporated at 43 °C under a stream of argon for 2 h, the vials were left at 43°C for further evaporation overnight. The mixtures were then heated to 80°C, stirred for 1 h and finally left to cool down to room temperature.

Carrier solution: Polyvinylalcohol (PVA, M ≈ 35.000, Sigma-Aldrich) was dissolved in MilliQ water under vigorous stirring at 80 °C (reflux). After cooling down to room temperature, glycerol was added to this solution and the mixture was stirred for at least 30 min to obtain 3 wt% PVA in a 1:1 solution of MilliQ water and glycerol.

### Preparation of the liquid crystal microspheres

The microspheres were generated using a microfluidic system designed for focusing flow technique^33^ (Fig. 1a). The cholesteric mixture (main stream) and the carrier solution (focusing stream) were pumped into a microfluidic device through its inlets using a Fluigent MFCS-Flex pressure control panel. The microsphere emulsion was collected via the outlet into a container. Microsphere formation for the required diameter was realized by adjusting the pressure ratios between the main and focusing streams. The entire process was monitored using a Labsmith SVM340 inverted microscope. Details of the design of the microfluidic device and the experimental parameters for the chip preparation are presented in Fig. S3 and Fig. S4.

### Preparation of the liquid crystal microbeads

For the polymer-stabilised microbeads, 4.7 wt% of the reactive mesogen RMS 257 (Merck) and 0.2 wt% of the photoinitiator Irgacure 379 (Ciba) (Fig. S1) were dissolved in dried dichloromethane and mixed with E7. Afterwards this solution was added to 3.9 wt% of the chiral dopant **1**-(R), mixed thoroughly, the dichloromethane was evaporated at 43 °C under a stream of argon for 2h and the vial was left at 43 °C for further evaporation overnight. The mixture was then heated to 80 °C, stirred for 1h and finally left to cool down. The preparation and storage of the polymerisable mixture was done in the dark.

For polymerization, about 20 μL of polymerisable microspheres were deposited on a microscope glass slide. After formation of a superstructure of microspheres, the sample was irradiated with a Spectroline long wave pencil lamp (2 mW/cm^2^) for 20 min.

### Optical characterization

Microsphere arrays were observed under a polarized optical BX51 microscope from Olympus, equipped with an Olympus DP73 digital camera using a UV cut-off filter (λ > 400 nm). For the images under circular polarized light, a U-TP137 quarter wave plate was used in the path of the light coming from the microscope. For photo-activation of the homogeneous superstructure of photo-responsive microspheres the UV light produced by the microscope halogen light source was sufficient, and no other external UV source was used. For photo-activation of the switchable droplets in the mixed superstructures, the samples were irradiated with a Hoenle bluepoint LED lamp (λ  = 365 nm, 350 mW/cm^2^).

## Additional Information

**How to cite this article**: Aβhoff, S. J. *et al.* Superstructures of chiral nematic microspheres as all-optical switchable distributors of light. *Sci. Rep.* **5**, 14183; doi: 10.1038/srep14183 (2015).

## Supplementary Material

Supplementary Information
