# Supplementary material for: Superstructures of chiral nematic microspheres as all-optical switchable distributors of light
Source: Sci Rep. 2015 Sep 24;5:14183. doi: 10.1038/srep14183 (PMC4585848; doi:10.1038/srep14183)
Supplement: Supplementary Information [file srep14183-s1.pdf]

Superstructures of chiral nematic microspheres as all-optical switchable distributors of light

Sarah J. Aβhoff[1], Sertan Sukas[2], Tadatsugu Yamaguchi[1], Catharina A. Hommersom[1], Séverine Le Gac[2]*, and Nathalie Katsonis[1]*

[1] Laboratory for Biomolecular Nanotechnology (BNT), MESA+ Institute for Nanotechnology, University of Twente, PO Box 217, 7500 AE Enschede, The Netherlands.

[2] BIOS, Lab on a Chip Group, MESA+ Institute for Nanotechnology and MIRA Institute for Biomedical Engineering and Technical Medicine, University of Twente, PO Box 217, 7500 AE Enschede, The Netherlands.

*Correspondence to: s.legac@utwente.nl, n.h.katsonis@utwente.nl

Table S1: Size distribution of the microspheres

Microsphere doped with	Mean radius [µm]	Standard deviation [µm]	Diameter [µm]
1	81.3	3.0	162.6 +/- 6.0
2-(S)	78.0	3.3	146.0 +/- 6.6
2-(R)	82.0	2.8	164.0 +/- 5.6
2-(S) and diacrylate	86.0	5.1	172.0 +/-10.2

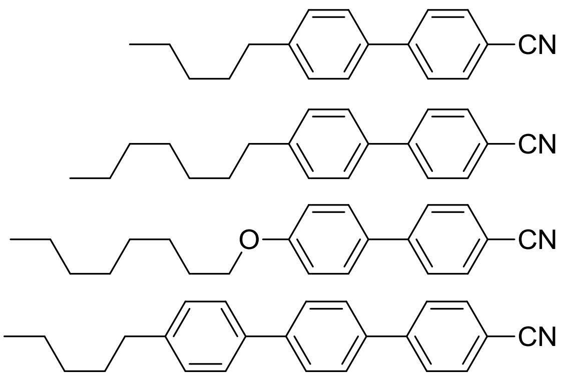

Nematic liquid crystal (E7)

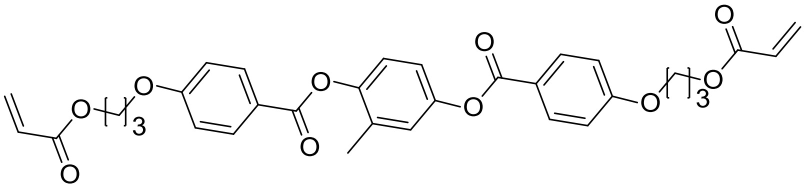

Reactive Mesogen

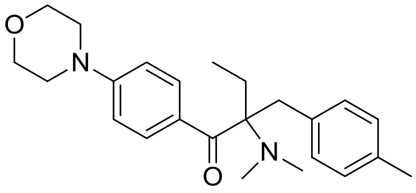

Photoinitiator (Irgacure 379)

Figure S1: Nematic liquid crystal host E7, the reactive mesogen and the photo-initiator used to prepare the chiral nematic liquid crystals used for the study.

Fabrication of the microfluidic device. The devices were fabricated from silicon/glass using standard micromachining processes. As a first step, the pattern of the fluidic channels was defined on the 500 µm thick, 4 inch silicon (Si) wafer via standard UV photolithography. Then 250 µm deep channels were etched using deep reactive ion etching (DRIE) into the Si wafer followed by the removal of the photoresist with oxygen plasma treatment and subsequent nitric acid (HNO_3) cleaning steps. After this, the access holes for the interfacing of the fluidic channels to the outside world were defined on the backside of the Si wafer via photolithography prior to the through etching of the wafer by another DRIE step, while the front side of the wafer was protected with a foil. Next, the processed Si wafer and a blank glass wafer (Borofloat) were cleaned thoroughly in piranha solution (3:1 volumetric ratio of H_2SO_4:H_2O_2) and pre-bonded followed by an anodic bonding step to create the permanent bond. As a last step, the stack was diced into individual microchips.

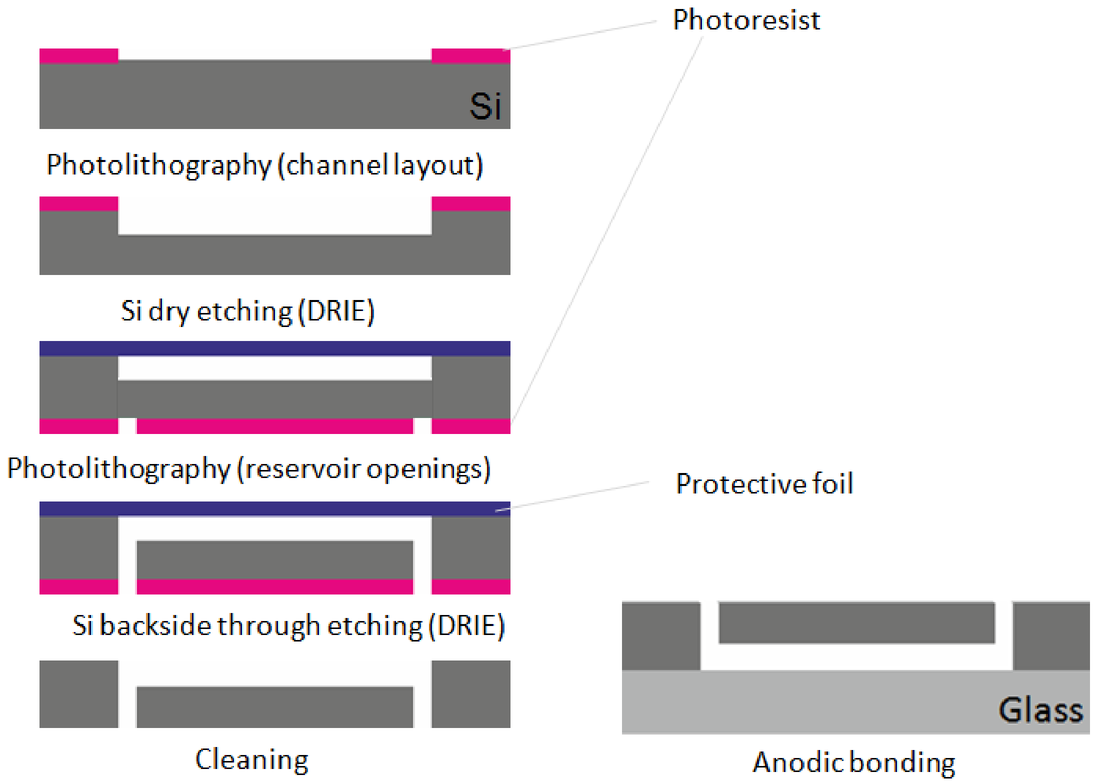

Figure S2: Overview of the fabrication process flow

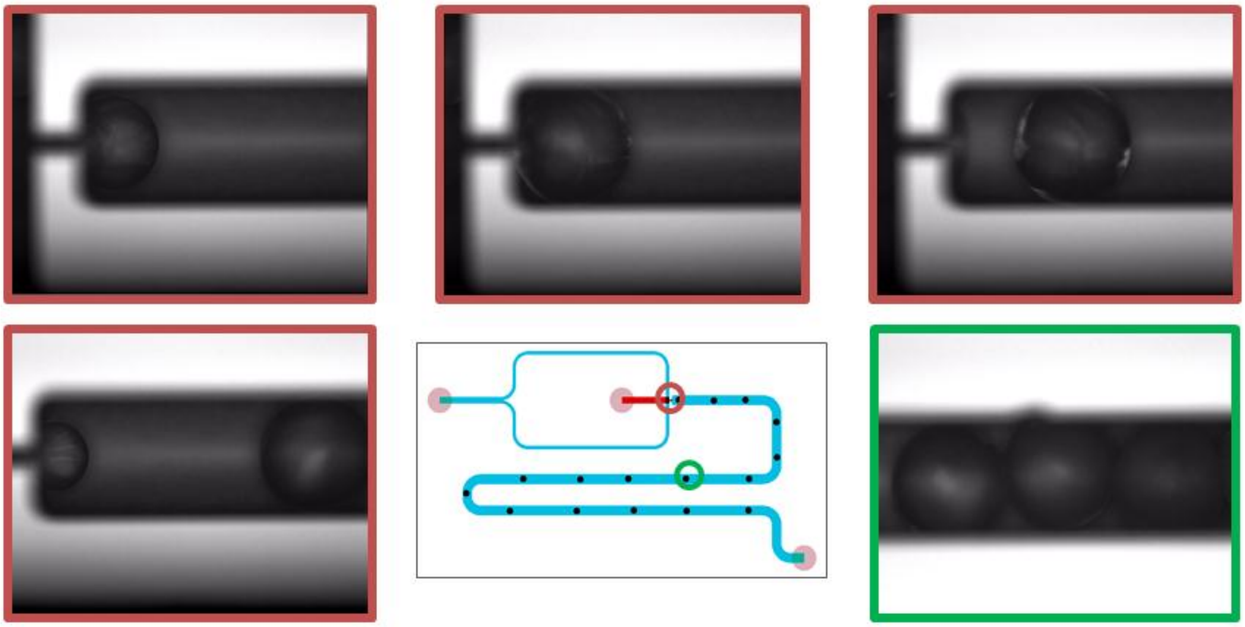

Figure S3: Chip design and pictures taken during generation of the microspheres.
